# *SCN4A* as modifier gene in patients with myotonic dystrophy type 2

**DOI:** 10.1038/s41598-018-29302-z

**Published:** 2018-07-23

**Authors:** Anna Binda, Laura V. Renna, Francesca Bosè, Elisa Brigonzi, Annalisa Botta, Rea Valaperta, Barbara Fossati, Ilaria Rivolta, Giovanni Meola, Rosanna Cardani

**Affiliations:** 1 School of Medicine and Surgery, University of Milano Bicocca, Monza, Italy; 2 Laboratory of Muscle Histopathology and Molecular Biology, IRCCS-Policlinico San Donato, Milan, Italy; 3 Department of Neurology, IRCCS Policlinico San Donato, San Donato Milanese, Milan, Italy; 4 Department of Biomedicine and Prevention, Tor Vergata University of Rome, Rome, Italy; 5 Research Laboratories, IRCCS Policlinico San Donato, Milan, Italy; 6 Department of Biomedical Sciences for Health, University of Milan, Milan, Italy

## Abstract

A patient with an early severe myotonia diagnosed for Myotonic Dystrophy type 2 (DM2) was found bearing the combined effects of DM2 mutation and Nav1.4 S906T substitution. To investigate the mechanism underlying his atypical phenotype,whole-cell patch-clamp in voltage- and current-clamp mode was performed in myoblasts and myotubes obtained from his muscle biopsy. Results characterizing the properties of the sodium current and of the action potentials have been compared to those obtained in muscle cells derived from his mother, also affected by DM2, but without the S906T polymorphism. A faster inactivation kinetics and a +5 mV shift in the availability curve were found in the sodium current recorded in patient’s myoblasts compared to his mother. 27% of his myotubes displayed spontaneous activity. Patient’s myotubes showing a stable resting membrane potential had a lower rheobase current respect to the mother’s while the overshoot and the maximum slope of the depolarizing phase of action potential were higher. These findings suggest that *SCN4A* polymorphisms may be responsible for a higher excitability of DM2 patients sarcolemma, supporting the severe myotonic phenotype observed. We suggest *SCN4A* as a modifier factor and that its screening should be performed in DM2 patients with uncommon clinical features.

## Introduction

Myotonic dystrophy type 2 (DM2) is caused by a CCTG repeat expansion in intron 1 of the *CNBP* gene located on chromosome 3q21^1,2^. The mutant RNA transcripts containing CCUG repeats alter the activity of specific RNA binding proteins involved in alternative splicing regulation. The missplicing affects, among others, the gene *CLCN1*encoding for the skeletal muscle chloride channel causing reduction of the chloride conductance, increased sarcolemmal electrical excitability, leading to myotonia^3^. Usually myotonia is mild and inconsistent in DM2, even by electromyography^4^. However, an association between DM2 patients with prominent myotonia and mutations on *CLCN1* or *SCN4A*, encoding for the α subunit of the skeletal muscle sodium channel Nav1.4, have been found. The additive effects of *CLCN1* missplicing and *CLCN1* or *SCN4A* mutation cause an atypical DM2 phenotype characterized by severe and early myotonia^5–12^. It may be of interest to recall that in non dystrophic myotonia the presence of concomitant mutations on *CLCN1* and *SCN4A* has been reported^13^ and variants of *CLCN1* gene modified the clinical and electrophysiological phenotype of *SCN4A*-mutated patients^14^. In our study, we investigated a DM2 patient with severe and early onset myotonia without mutations in *CLCN1* or *SCN4A* genes. Nevertheless, a c.2717G > C base exchange in exon 14 of the *SCN4A* gene predicting an S906T substitution was identified. This variant is considered a polymorphism. To investigate the atypical DM2 phenotype of our proband, the combined effects of DM2 mutation and S906T substitution in Nav1.4 have been studied performing whole-cell voltage- and current-clamp in myoblasts and myotubes derived from muscle biopsy of the patient. Results have been compared to those obtained in muscle cells derived from his mother, who is also affected by DM2, but does not present the S906T polymorphism.

## Results

### Patients

Patient 1. The proband, a 30 years old man, was admitted to the Department of Neurology of the IRCCS Policlinico San Donato because of muscular stiffness and grip myotonia since the age of 12. The patient complained difficulties in fine finger movements and diffuse muscle stiffness especially when he woke up in the morning. These symptoms progressively worsened over time and with cold, while improved with repetitive movement. Neurological examination revealed normal muscle strength (grade 5 MRC in all muscles tested) and tone, a lid lag, eyelid and mild tongue myotonia. Severe grip myotonia was evident, with a positive warm-up phenomenon. Deep tendon reflexes were normal. The EMG showed diffuse signs of myotonic discharges in all muscles tested. EKG, Holter electrocardiographic recordings and echocardiogram were normal. Laboratory studies demonstrated normal electrolyte, urea, creatinine, and lactate dehydrogenase levels. CPK was 231 U/L (normal values < 190 U/L). AST (56 U/L; normal values < 41 U/L) and GGT (106 U/L; normal values 8–61 U/L) were mild increased. Lipid profile showed high levels of total (260 mg/dl; normal values < 200 mg/dl) and LDL (174 mg/dl; normal values < 159 mg/dl) cholesterol and triglycerides (356 mg/dl; normal values < 200 mg/dl) with the evidence of hepatic steatosis at the abdomen ultrasound.

Patient 2. The proband’s mother was 64 years old when she was admitted to our department. She complained muscular stiffness, difficulties in climbing stairs and rising from the squatting position since the age of 57. Bilateral cataracts surgery before 60 years old and asthma were referred. Neurological examination revealed normal muscle strength except for facial mimetic muscles, neck flexors (grade 4 MRC), shoulders abductors (grade 4 MRC), brachial biceps (grade 4 MRC) and hip flexors (grade 4 MRC). Deep tendon reflexes were uniformly diminished. Clinical myotonia was absent. Muscle tone was normal except for bilateral gastrocnemius hypertrophy. The EMG study showed myotonic discharges in all muscles examined, but no myopathic changes. EKG, Holter electrocardiographic recordings, and echocardiogram were normal. Routine laboratory studies were normal except for serum creatine levels (229 U/L; normal values < 190 U/L) and mild hypercholesterolemia.

Neurological examination of the father resulted negative and for this reason he refused to undergo further investigations.

### Biomolecular diagnosis

Both in the proband and his mother, FISH in combination with MBNL1 immunofluorescence on muscle sections revealed the presence of nuclear accumulation of toxic RNA and of MBNL1 protein as commonly observed in DM2 patients (data not shown).

The genetic analysis on blood DNA confirmed the diagnosis of DM2 showing the presence of an expansion of about 1000–2500 CCTG repeats in the *CNBP* gene in both patients.The proband’s father resulted negative for DM2.

### Muscle histology and alternative splicing and quantification of the CLCN1 mRNA

**Figure 1:**
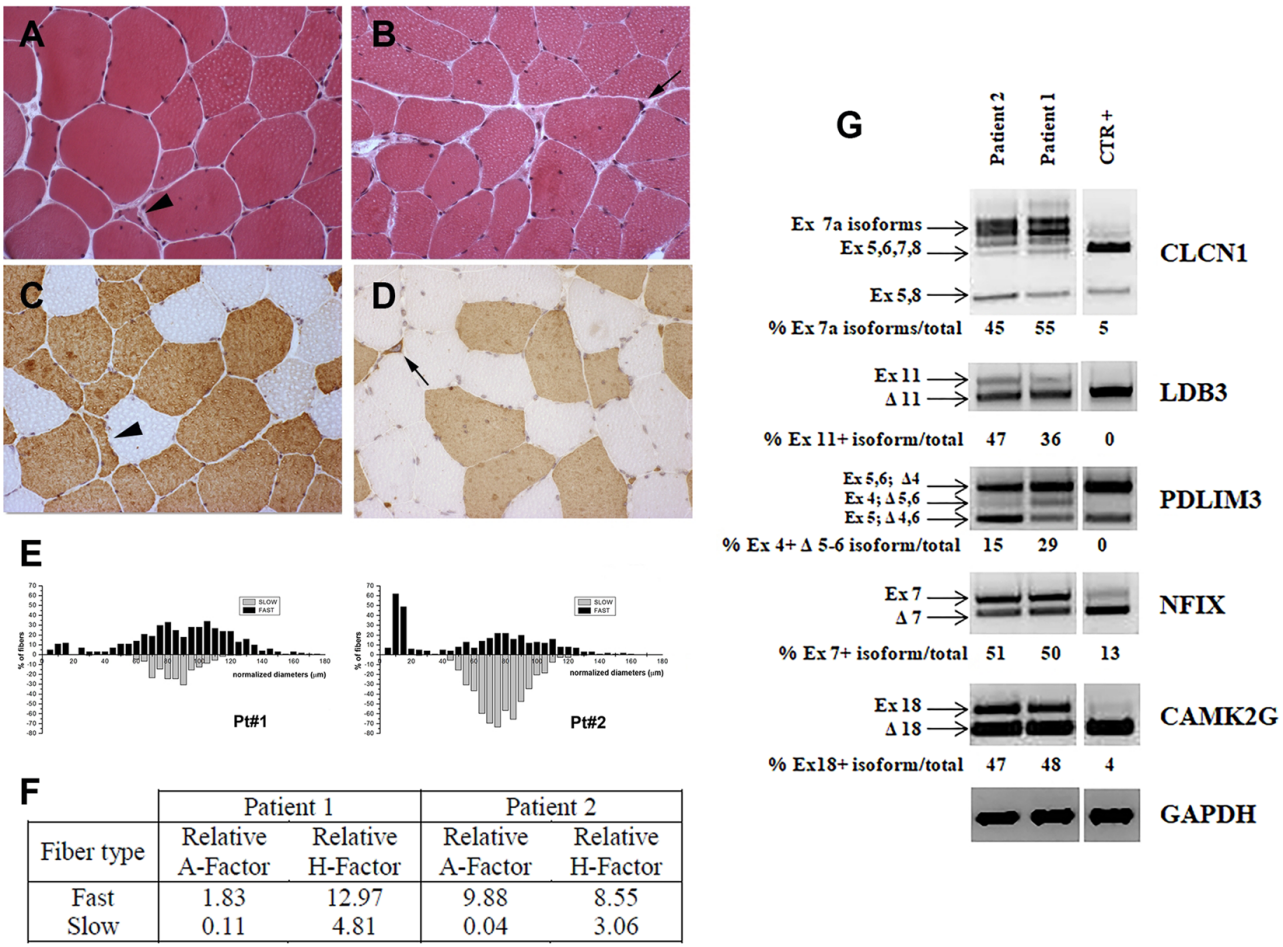
Histopathology, immunocytochemistry and splicing analysis. (**A–D**) Histopathological analysis of biceps brachii biopsy obtained from patient 1 (**A**,**C**) and patient 2 (**B**,**D**). Haematoxylin and eosin (**A**,**B**) demonstrates a variation in fiber size with atrophic fibers (arrowhead), central nuclei and pycnotic nuclear clumps (black arrow) in both patients. Immunostaining (**C**,**D**) shows that atrophic fibers (arrowhead) and nuclear clumps (black arrow) are fast myosin positive. (**E**) Metahistograms obtained from the analysis of muscle fiber diameters in patient 1 and patient 2 on sections immunostained for MHCf or MHCs. Type 2 fiber atrophy is present in both patients but more evident in patient 2. Marked hypertrophy of both type 1 and type 2 fibers is particularly evident in patient 1. (**F**) Table shows the relative atrophy (**A**) or hypertrophy (**H**) factor in patient 1 and patient 2. (G) Panel showing the RT-PCR splicing analysis of skeletal muscle chloride channel (*CLCN1*), LIM Domain Binding 3 (*LDB3*), α-actinin-associated LIM protein 3 (*PDLIM3*), nuclear factor 1X (*NFIX*), calcium/calmodulin-dependent protein kinase II gamma (*CAMK2G*) genes in muscle from patients 1 and 2 and in a healthy subject (CTR). Due to the presence of samples not related to this study, the figure panel is composed by cropped images originating from the same gel. Details on how RT-PCR alternative splicing analysis was performed is reported in Materials and Methods. Alternatively spliced exons analyzed for each gene and the relative percentage of abnormal isoforms are indicated.

Both in the proband and his mother, routine histological and histochemical staining of muscle sections showed the characteristic pathological features of DM2. Increased fiber size variation, internalized nuclei and many small fibers with nuclear clumps were present (Fig. 1A–D). Immunostaining for MHCf or MHCs allowed to detect and measure fibers with a diameter smaller than 20 μm and to observe that the majority of marked atrophy, preferential central nucleation and nuclear clumps were evident in type 2 fibers (Fig. 1C,D). In patient 1, the metahistograms based on data obtained from the analysis of muscle fiber diameters revealed a preferential type 2 fiber atrophy and an evident hypertrophy of both fiber types (Fig. 1E). Indeed, in this patient a marked increase of the relative hypertrophy factor of type 2 fibers has been calculated (Fig. 1F). In patient 2, the metahistogram of type 2 fibers had a bimodal distribution due to the presence of both normal-sized and numerous atrophic fibers with abundant type 2 nuclear clump fibers (Fig. 1E). Hypertrophy of both type 1 and type 2 fibers was also evident (Fig. 1F).

As expected, both patients showed an altered splicing pattern of the genes analysed as commonly observed in DM2 patients. (Fig. 1G).

QRT-PCR analysis to quantify the expression levels of the *CLCN1* transcripts in skeletal muscle showed a considerable downregulation of this gene in DM2 patients compared to control (n = 1). Levels of *CLCN1* mRNA were lowered to 47% and 57.5% in our proband and his mother samples respectively, relative to control (data not shown).

### Molecular genetic analysis

Direct sequencing of the entire coding region of *CLCN1* gene in patient 1 and in his parents did not reveal the presence of mutations. However, genetic analysis of *SCN4A* gene identified two heterozygous missense polymorphisms in the proband: a c.2717G > C base exchange in exon 14 (refSNP rs41280102; NM_000334.4:c.2717G > C; NP_000325.4:p.Ser906Thr) and the c.4146A > G variant in exon 23 (refSNP rs2058194; NM_000334.4:c.4126A > G; NP_000325.4:p.Asn1376Asp). The latter one is a known polymorphism of the SCN4A gene^5^, whereas the c.2717G > C base exchange is considered a “likely benign” polymorphism as the encoded serine to threonine aminoacid substitution can affect the functional properties of the Nav1.4 channel^6^. Segregation analysis in the family indicated that the c.2717G > C variant allele was paternally inherited.

### Functional analysis of myoblasts sodium current and myotubes action potentials (APs)

**Figure 2:**
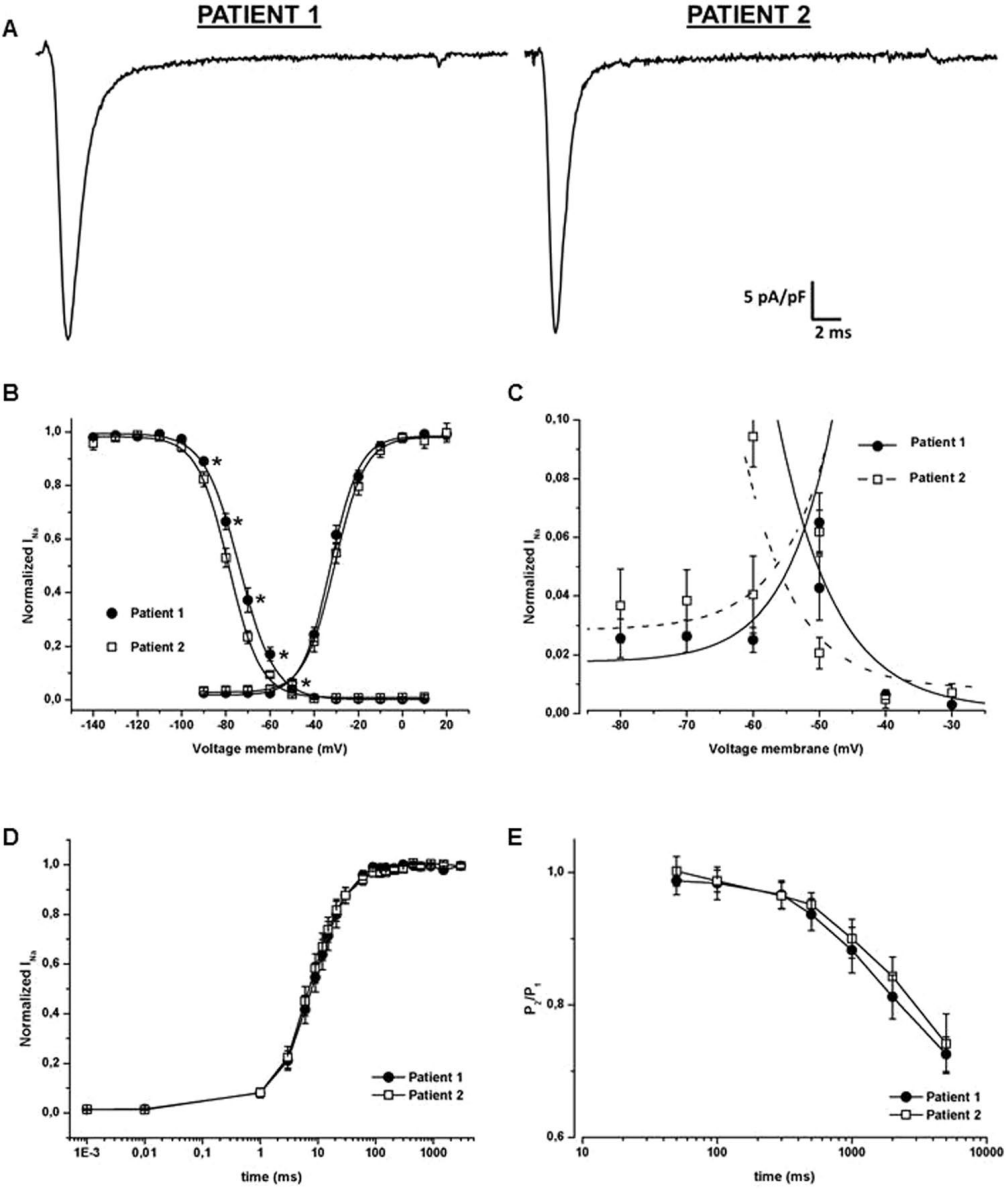
Functional characterization of sodium current in myoblasts derived from the two patients. (**A**) Representative peak currents evoked by a stimulus of −10 mV and normalized by cell capacitance; sodium current density was similar between patient 1 (proband) and patient 2 (mother). (**B**) Steady-state activation (right) and fast inactivation (left) curves. No significant difference was found in the conductance-voltage relationships of the two patients (n = 13 in patient 1 and n = 9 in patient 2). A significant shift of about 5 mV in the positive direction was instead observed considering the steady-state fast inactivation curve of patient 1 (filled symbols, n = 16) compared to patient 2 (empty symbols, n = 9). (**C**) Enlargement of the portion of the overlapping curves of panel B to highlight the increased window current. (**D**) Recovery from fast inactivation. Peak current amplitude was normalized to the fully recorded current and plotted as function of time after imposition of the conditioning pulse. Overlapping curves were obtained for patients 1 (n = 17) and 2 (n = 14). (**E**) Entry into slow inactivation. Time dependence of the onset of slow inactivation was measured using a two pulse protocol, the resulting P2/P1 ratio was plotted against the P1 duration. The two obtained curves did not show any significant differences among the proband (n = 8) and his mother (n = 10). In the figure *p < 0.05.

Similar sodium current amplitude evoked by a −10 mV stimulus was found in myoblasts derived from patient 1 and patient 2, as demonstrated by the current density and the cell capacitance (Fig. 2A, Table 1). The decay of the inactivation of the peak current revealed comparable slow time constant (τ_slow_) but different fast one (τ_fast_), being the proband’s one slower than his mother’s (Table 1).

**Table 1:** Parameters recorded from myoblasts derived from patient 1 and 2.

		Patient 1	Patient 2
Cell capacitance (pF)		53.11 ± 6.1 (n = 100)	52.23 ± 5.7 (n = 63)
Current Density (pA/pF)		−35.45 ± 5.5 (n = 100)	−34.15 ± 6.05 (n = 63)
τ_slow_ (ms)		7.44 ± 0.9 (n = 85)	6.60 ± 0.7 (n = 50)
τ_fast_ (ms)		0.93 ± 0.06 (n = 100)* (p = 0.018)	0.76 ± 0.04 (n = 63)
Steady-state activation	V_1/2_ (mV)	−32,87 ± 1 (n = 13)	−30,69 ± 1,1 (n = 10)
	k	6,5 ± 0,4 (n = 13)	6,7 ± 0,8 (n = 10)
Steady-state fast inactivation	V_1/2_ (mV)	−73,86 ± 1,3* (n = 8, p = 0.0019)	−78,82 ± 1 (n = 9)
	k	7,7 ± 0,2 (n = 8)	7,17 ± 0,3 (n = 9)

The number of cells considered for the τ_slow_ is smaller compared to the one of the other parameters as we eliminated the negative values that came out from the fitting analysis (in both the patients this elimination regarded about 15% of cells). The number of independent experiments were 17 for patient 1 and 15 for patient 2. *p < 0,05

While the activation curves were overlapping, the voltage dependence of steady-state fast inactivation of patient 1 was 5 mV significantly shifted towards more positive potentials compared to patient 2 (Fig. 2B and Table 1 for V_1/2_ and k values).This right shift affected the window current, changing its peak from about −56.5 mV to −52.2 mV and causing a higher channel availability in the voltages range between −80 and −30 mV in the proband compared to the mother (Fig. 2C). Finally, the time course of the recovery from fast inactivation as well as the time dependence of the onset of slow inactivation did not show any differences between the two patients (Fig. 2C,D).

**Figure 3:**
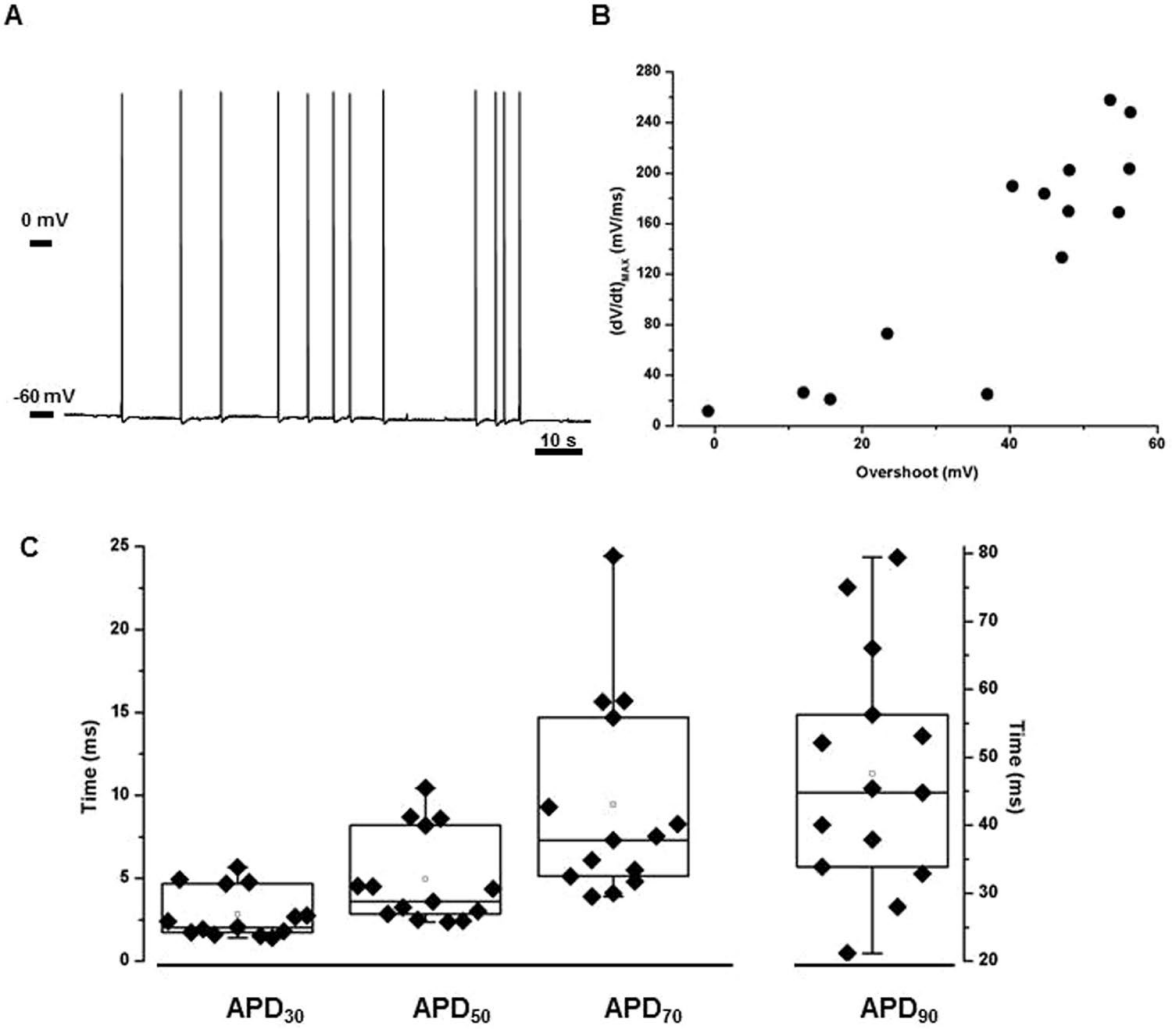
Spontaneous activity in myotubes from patient 1. (**A**) Representative recording of spontaneous APs. When voltage membrane was unstable, firing frequency was not homogeneous either among the different cells and also within a single cell recording. (**B**) Uprising phase slope/overshoot relationship. Although the characteristics of spontaneous APs lay in an heterogeneous range of values, (dV/dt)_MAX_ measured during in the phase 0 of an AP could be related to the overshoot of the same AP (n = 14). (**C**) Variability of APD_30_, APD_50_, APD_70_ and APD_90_ in spontaneous action potentials (n = 14). Each filled symbol represents a single myotubes, box plots contain data between percentile 25 and 75, the straight line and the white dot sets the median and the mean values respectively.

The resting membrane potential values (V_rest_) recorded in myotubes derived from patient 2 were stable and homogeneous, and averaged at −39.88 ± 2.9 mV (n = 70), consistent with previous reported data^7^. Surprisingly, in myotubes from patient 1 a dual population was observed: while the 73.4% of cells had a resting potential comparable to patient 2 (−41.54 ± 3.22 mV, n = 72), the remaining 26.6% of the cells were significantly hyperpolarized (V_rest_ = −54.41 ± 5.3, n = 27, p < 0.001) and characterized by the presence of spontaneous APs that could not be easily framed. Indeed, the spontaneous activity had not a reproducible frequency (Fig. 3A) and the APs overshoot was different almost for every cell recorded. However, although the slope of the APs uprising phase was also specifically diverse for each myotube considered, the relationship between the (dV/dt)_MAX_ and overshoot was maintained (Fig. 3B). The APD_30_, APD_50_, APD_70_ and APD_90_ similarly confirmed the heterogeneity of the phenomenon (Fig. 3C).

**Figure 4:**
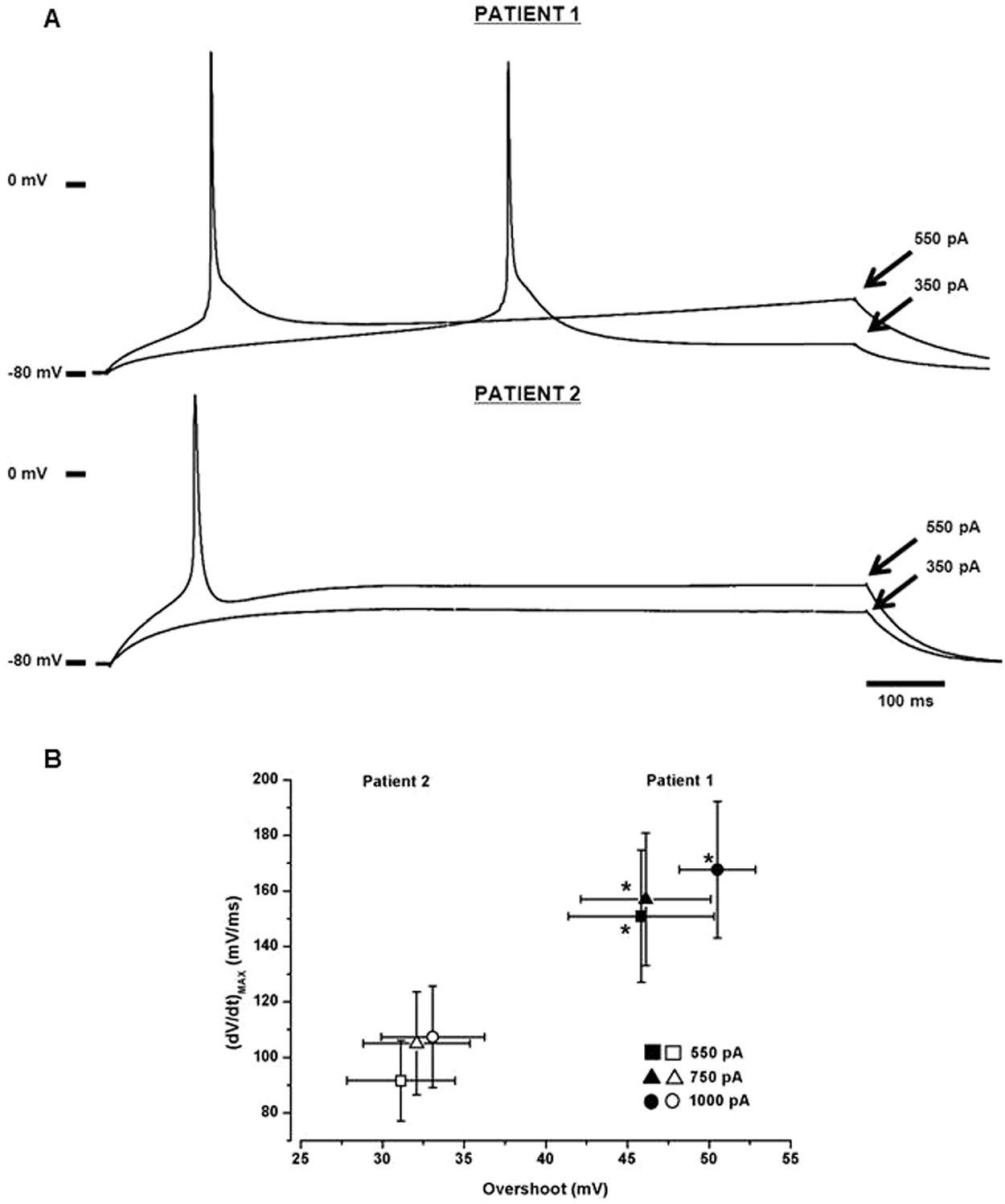
Myotubes action potential characteristics. (**A**) The rheobase current was significantly reduced in patient 1 compared to patient 2. Indeed, representative tracks show that, in the first case, a 350 pA stimulus was sufficient to evoke an AP, while the patient 2 a 550 pA current was needed in. (**B**) Uprising phase slope/overshoot relationship. The maximum voltage value reached by evoked APs was higher and the (dV/dt)_MAX_ was faster in patient 1 (n = 34) compared to patient 2 (n = 30) in correspondence of every current stimulus applied. Here for seek of clarity, only three stimuli were shown. In the figure *p < 0.05.

In myotubes from patient 1showing a stable resting membrane potential, the rheobase current was significantly lower compared to the ones of patient 2 (352.37 ± 80.7 pA, n = 59 and 578.60 ± 134.1 pA, n = 57, respectively; p < 0.001; Fig. 4A) while the APs overshoot as well as the maximum slope of the uprising phase ((dV/dt)_MAX_) were significantly higher in patient 1 compared to patient 2, for all the injected stimuli tested (Fig. 4B, only three injected stimuli shown).

Considering the duration of action potentials, while in patient 1 APD_30_ was reduced respect to patient 2, APD_90_ was instead increased. APD_50_ and APD_70_ were comparable between the patients (Table 2).

**Table 2:** Action potential duration at 30, 50, 70 and 90% of repolarization (APD_30_, APD_50_, APD_70_ and APD_90_ respectively) measured in myotubes derived from patient 1 and patient 2.

		Injectedcurrent (pA)		
		550	750	1000
APD_30_ (ms)	Patient 1	2.64 ± 0.4* (n = 20)	2.75 ± 0.3* (n = 21)	2.64 ± 0.3* (n = 21)
	Patient 2	3.30 ± 0.3 (n = 21)	3.19 ± 0.2 (n = 26)	3.16 ± 0.2 (n = 34)
APD_50_ (ms)	Patient 1	4.33 ± 0.6 (n = 20)	4.44 ± 0.5 (n = 21)	4.29 ± 0.5 (n = 20)
	Patient 2	4.96 ± 0.5 (n = 22)	4.90 ± 0.4 (n = 27)	4.82 ± 0.3 (n = 35)
APD_70_ (ms)	Patient 1	7.57 ± 1.0 (n = 20)	7.63 ± 1.0 (n = 21)	6.87 ± 0.8 (n = 21)
	Patient 2	7.72 ± 0.9 (n = 22)	7.68 ± 0.8 (n = 27)	7.48 ± 0.6 (n = 35)
APD_90_ (ms)	Patient 1	26.20 ± 6.4* (n = 20)	24.00 ± 6.6* (n = 21)	17.09 ± 2.4* (n = 21)
	Patient 2	15.04 ± 2.3 (n = 22)	15.15 ± 2.2 (n = 27)	14.13 ± 1.7 (n = 34)

The number of independent experiments were 5 for both the patients. *p < 0,05

Finally, in petri dishes of the proband’s derived cells, myotubes contraction lasting for several minutes were sometimes observed but could not be properly described due to their occasional nature.

## Discussion

Recent studies highlighted how digenic interactions in neuromuscular disorders may be more frequent than previously estimated. The evidence is that many genetic factors can contribute to the phenotype variability. Concerning myotonic dystrophies, the co-segregation of DM2 mutation with mutations in *CLCN1* or *SCN4A* genes were linked to unusual clinical findings^8–12^.

The case described here supports the importance of considering the interaction among multiple genetic variants in determining the patient phenotype, disease mechanisms and modality of inheritance.

The proband is a patient affected by a maternally transmitted DM2 with an early and severe myotonia in absence of associated mutations in *CLCN1* gene. He also carried a c.2717G > C base exchange in *SCN4A* gene, predicting an S906T substitution considered a benign polymorphism with a frequency of about 1.5% in the European population according to the Genome Aggregation Database (gnomAD) and described in patients with neurological diseases not related to channelopathies or with either hypo- or hyperkalemic periodic paralysis with a frequency of about 5%^6^. This variant allele was inherited by the asymptomatic father and the proband clinical phenotype appeared more severe than that of his mother, who lacks the *SCN4A* polymorphism. As a consequence of the more severe myotonia, the muscle of the proband showed a higher fiber hypertrophy factor of both fiber types than those observed in the mother. Moreover, in agreement with previously reported data^9^ the expression levels of the *CLCN1* gene were similarly reduced in skeletal muscle of our DM2 patients compared to healthy subject suggesting that the more severe myotonia in our proband is not related to a lower *CLCN1* mRNA expression compared to his mother. For these reasons, we have hypothesized that the association of DM2 mutation with the Nav1.4 S906T variant may have an additive effect at a pathological level and could eventually explain the observed atypical severe myotonia. To address this issue, we compared the properties of the sodium current recorded in myoblasts and the features of the action potential in myotubes derived from the proband and from his mother skeletal muscle biopsies.

The S906 residue is located within the loop joining the second and the third transmembrane domains of the Nav1.4 channel, an important region for slow inactivation. In tsA201 cells, this variant resulted in a reduced sodium current density and in a slowdown both of the entry into and of the recovery from the slow inactivation compared to the WT protein^6^. Unlike these data, we observed a comparable sodium current density and a similar development of the slow inactivation between myoblasts derived from the proband and from the mother, harboring the Nav1.4 S906T variant and WT respectively. Myoblasts are, however, definitively a more complex cellular system than tsA201 cells, hence one could expect that data obtained in the two different cellular systems do not overlap.

Although the τ_slow_ did not differ in the two patients, the τ_fast_ was significantly slower in myoblasts derived from the proband, suggesting a higher contribution of the inward current in the early phase of the action potential. Furthermore, the steady-state fast inactivation curve of the proband had a significant +5 mV shift compared to his mother. As this last curve gives an indication of the fraction of activable Na^+^ channels^15^, its depolarizing shift may be responsible for a higher number of channel available to open in the voltage range between −80 mV and −30 mV. Together, it might affect the window current, a non-inactivating sustained sodium current which derives from the overlap between steady-state activation and inactivation curves. Other *SCN4A* mutations described in patients affected by myotonia caused an increased window current, and this was proposed as causative mechanism for the tissue hyperexcitability^16,17^. Collectively, these data may suggest that, in DM2 derived myoblasts, the presence of Nav1.4 S906T polymorphism lead to a “gain-of-function” mechanism. Indeed, impaired sodium inactivation due to an increased decay time and or to a positive shift of the voltage-dependence of steady-state inactivation are common features observed in several cases of sodium channel myotonia^16^.

With regards to myotubes, a consistent group of cells of the proband showed a spontaneous firing activity. This feature is uncommon^18^, as it is the spontaneous contraction activity observed in some spots of the petri dishes in absence of any control of temperature and/or CO_2_ level or nerve-derived factor for muscle differentiation^7,19^. Even if the strong heterogeneity within this proband-exclusive myotubes subpopulation prevented us from framing it, we would like to emphasize that a self-sustained trains of action potentials persisting beyond the termination of a current stimulus is the reported model correlating to myotonia^16^.

Considering the myotubes having a stable resting membrane potential, data recorded in the proband compared to the mother cells revealed a reduction of the rheobase current and increased values in the amplitude vs rate of rise of the action potentials depolarizing phase. These results may agree with the predicted increased membrane excitability observed in the proband’s myoblasts.

Finally, a prolonged APD_90_ in the proband action potentials compared to the mother point out that the total duration of each single electrical event was increased in the former. This prolongation may be justified by the non-physiological entrance of positive charges due to the rightward shift in the steady-state fast inactivation and the larger window current in a range of voltages related to the final phase of an action potential that should oppose to membrane hyperpolarization.

Since the S906T polymorphism, here passed by the proband’s father, is present in the general healthy population, we assume that it cannot cause myotonia by itself, nevertheless we are more prone to believe in its role as a modifier. Recently, data on a group of related and unrelated patients affected by hyperkalemic periodic paralysis (HyperPP) carrying the S906T polymorphism in addition to a mutation (I692M) in Nav1.4 channel suggested that the coexistence of the two variants, in that case on the same allele though, somehow exacerbates the paralytic phenotype^20^. When expressed in heterologous system, the compound mutations induced a negative shift in the conductance-voltage relationship and an enhanced close-state inactivation^20^. As a whole, these characteristics go towards a shutdown of the current, which justifies the muscular weakness typical of the pathology in question. In the case presented in this study, instead, the presence of the S906T substitution promote the sodium current, increasing the excitability of the substrate, a scenario consistent with the myotonic phenotype. Analogously to the study reported on HyperPP, since DM2 manifestations were early and more relevant in our proband compared to the mother, we may propose that the S906T polymorphism aggravates the clinical appearance of the disease. Therefore, in accordance to our previous study^21^, we suggest that *SCN4A* gene should be classified as a modifying gene for DM2.

We are aware that this study presents some limitations: a skeletal muscle biopsy of the father could not be performed, therefore we could not provide data from his myoblasts and myotubes derived cells and, likewise, we could not provide data on control muscle cells since true control biopsies from healthy volunteers were not available for ethical reasons. However, we intended to evaluate the *SCN4A* variant role in a DM2 phenotype, thus to this aim the mother represents a valuable internal control of the proband.

In conclusion, the results of our study support that the presence of S906T polymorphism in Nav1.4 on a DM2 genetic background may promote a general increased cellular excitability, manifested with an uncommon severe and early onset myotonic phenotype. Thus, we suggest that, in cases of patients with an unusual phenotype, the genetic screening of *SCN4A* is worth to be performed. Moreover, a personalized therapy could take advantages of the identification and of the functional characterization of modifying factors. Indeed, in our proband, mexiletine treatment, that is the election choice drug in cases of myotonia, was ineffective. Mexiletine is a class 1b antiarrhythmic drug but could also prevent or contrast myotonic discharges since it induces a use-dependent block of Nav1.4 channels. However, the drug has a higher affinity for the inactivated than for the close or open state of sodium channels^22^. Thus, the less mexiletine efficacy in the proband could be explained by the observed depolarized shift of the steady-state inactivation curve, which lead to a destabilization of the inactivation properties of the channel. Other pharmacological treatment are under investigation to identify a personalized therapy.

## Methods

### Patients

Two patients, the proband (patient 1) and his mother (patient 2), were clinically evaluated by a neurological examination and electromyography (EMG). EKG, Holter electrocardiographic recordings, echocardiogram, and blood tests were performed. Neurological examination of the proband’s father was also performed. Reports on other family members were obtained through the patients.

A biceps brachii muscle biopsy was taken from the proband at the age of 30 years and from his mother at the age of 64 years.

DM2 molecular diagnosis was performed by fluorescence *in situ* hybridization (FISH; see below) on muscle sections and by genetic test performed on blood DNA according to Valaperta *et al*.^23^.

This study was authorized by the Institutional Ethics Committee of the Local Health Unit (ASL MI2, Melegnano, Milan, Italy) and was conducted according to the principles expressed in the Declaration of Helsinki, the institutional regulation and Italian laws and guidelines. Written informed consent were obtained from the patients for all blood samples and muscle biopsies used in this study.

### Muscle histopathology and immunohistochemistry

Muscle tissues were fresh-frozen in isopentane cooled in liquid nitrogen. Histopathological analysis was performed on serial sections (8 µm) processed for routine histological or histochemical staining.

Serial transverse muscle cryostat sections 6 µm thick were cut for fast or slow myosin heavy chain (MHC) immunohistochemical staining (IHC) as previously reported^24^. Mouse monoclonal primary antibodies against MHCfast (MHCf; MY32 Sigma-Aldrich 1:400 in PBS + 2% BSA) and MHCslow (MHCs; NOQ7.5.4D 1:400 in PBS + 2% BSA) and a goat anti-mouse biotinylated secondary antibody (1:300 in PBS + 2% BSA) were used. Quantitative evaluation of fiber diameter was made as previously described^25^ with Scion Image (Scion Corporation) on images taken with a light microscope (Axio Imager M1, Zeiss) at 200x magnification. Muscle fibers size was assessed by measuring the “smallest fiber diameter”. Data were elaborated using Microcal Origin (Microcal Software Inc.). The metahistograms were normalized to normal mean diameter for men and women. Atrophy and hypertrophy factors were also calculated^25^.

### FISH and Muscleblind-like1 protein (MBNL1) immunofluorescence

FISH procedure, using RNA (CAGG)5-Texas red labeled probes (IDT), was carried out in combination with MBNL1 immunostaining on muscle sections as previously reported^24,26^. A polyclonal rabbit anti-MBNL1 (1:1000 in PBS + 2% BSA; gift from Prof. C.A. Thornton, University of Rochester, New York, USA) and an Alexa488-labeled goat anti-rabbit secondary antibody (5 µg/ml in PBS + 2% BSA; Molecular Probes) were used. DAPI nuclei staining was performed.

### Primary skeletal muscle cell cultures

The human satellite cells-derived myoblasts were isolated from patient 1 and patient 2 muscle biopsies as previously reported^16^. Myogenic purity and differentiative capability were evaluated^27^. Myoblasts were grown in HAM’s F10 medium (Sigma-Aldrich) supplemented with 15% FBS (EuroClone), 0.5 mg/ml albumin from bovine serum (BSA, Sigma-Aldrich), 0.5 mg/ml fetuin (Sigma-Aldrich), 0.39 g/ml dexamethasone (Sigma-Aldrich), 10 ng/ml epidermal growth factor (Sigma-Aldrich), 0.05 mg/ml insulin (Insulin aspart, NovoRapid), 3 mg/ml glucose, 100 U/mL penicillin and 100 μg/mL streptomycin (proliferative medium). Cells were grown in proliferative medium at 37 °C in a humidified 95% air/5% CO_2_ atmosphere. All cell populations used in this study had a myogenic purity higher than 90%. Myoblasts were allowed to grow until 80% confluence to initiate differentiation. The proliferative medium was replaced with differentiative medium consisting of Dulbecco Modified Eagle Medium (DMEM, Sigma-Aldrich) supplemented with 7% FBS, in presence of 100 U/ml penicillin and 100 μg/ml streptomycin.

### Alternative splicing analysis and qRT-PCR analysis

Alternative splicing of *CLCN1*, *LDB3*, *PDLIM3*, *NFIX* and *CAMK2B* genes was analyzed in muscle biopsy from patient 1 and 2 and in a healthy subject used as control (CTR). Total RNA was extracted from biceps brachii biopsies using TRIzol reagent (Life Technologies) according to manufacturer’s instructions. RNA quantity and quality was assessed using the NanoPhotometer NP80 (Implen) and an equal amount of RNA for each sample was retro-transcribed in complementary DNA by SuperScript III First-Strand Synthesis System for RT-PCR (Life Technologies). RT-PCR splicing analysis for *CLCN1* was performed using PlatinumTaq Polymerase (Invitrogen); analysis of *LDB3*, *PDLIM3*, *NFIX* and *CAMK2B* alternative splicing was performed using MyTaq Red Mix (Bioline); 100 ng of cDNA for each sample were used. Primers sequence and temperature of melting are: *CAMK2B Fw* 5′- CAGGAGACTGTGGAGTGTCTG-3′, Rev 5′-AGCGTCTTCATCCTCTATGGTGG-3′ (Tm 62 °C), *CLCN1* Fw 5′-GGTTGTCCTGAAGGAATACCTCAC-3′, Rev 5′-TCCTCTCCAGTAGTTCCGAACAG-3′ (Tm 60 °C), *LDB3* Fw 5′-GACTACCAGGAACGCTTCAACC-3′, Rev 5′-GACAGAAGGCCGGATGCTG-3′ (Tm 62 °C), *NFIX* Fw 5′-GAGCCCTGTTGATGACGTGTTCTA-3′, Rev 5′-CTGCACAAACTCCTTCAGTGAGTC-3′ (Tm 62 °C), *PDLIM3* Fw 5′-AGCCCATCCTTTCAAAATCAAC-3′, Rev 5′-AGAGCCATCGTCCACCATTC-3′ (Tm 58 °C). Total PCR products were electrophoretically resolved on 1.5% agarose gel or on 6% acrylamide gel for *CLCN1*and scanned on a ChemiDoc Universal Hood (Biorad); quantitative analysis were performed quantifying the intensity of each band with ImageJ software densitometry and calculating the proportions of abnormally spliced isoforms respect to the total amount of the isoforms.The expression level of *GAPDH* was used as housekeeping. Primers: *GAPDH* Fw5′-AGCCTCCCGCTTCGCTCTCT-3′ and *GAPDH* Rev 5′- GCCAGCATCGCCCCACTTGA-3′ (Tm 60 °C).

Total expression of *CLCN1* gene was evaluated by qRT-PCR on muscle biopsy from patient 1 and 2 and in a healthy subject used as control (CTR). The specific *CLCN1* TaqMan gene expression assay Hs00163961_m1 was used and *GAPDH* gene was chosen as housekeeping internal control (Hs02758991_g1) (Applied Biosystem). Each PCR reaction was performed in triplicate using the TaqMan Universal PCR Master Mix and the StepOne Plus Real Time PCR system (Applied Biosystems).

### Molecular genetic analysis

*CLCN1* and *SCN4A* genetic analysis were carried out on the proband and his parents blood samples for segregation analysis. NGS assay was designed for sequencing the entire *CLCN1* gene coding region (23 exons, NM_000083), including intron/exon junctions^5,28^. Analysis of *SCN4A* gene was performed by PCR amplification of highly purified genomic DNA, followed by automated bi-directional DNA sequencing of the entire *SCN4A* coding region (24 exons, NM_000334.4, primers available upon request). Sequencing included also the highly conserved flanking intronic sequence of the exon/intron splice junctions for all coding exons and 10 bases of intronic DNA surrounding each exon.

### Electrophysiological studies

Myoblasts were plated 24 hours in advance. Patch-clamp recordings were performed at RT using pipettes pulled to a 2–5 MΩ resistance (Model P-97, Sutter Instruments). Before starting whole-cell patch-clamp recordings, cellular medium was replaced with an extracellular solution containing (in mM) 140 NaCl, 5 KCl, 1.8 CaCl_2_, 1 MgCl_2_, 10 HEPES, 10 glucose (pH 7.4 with NaOH). Pipettes were filled with (in mM) 135 CsCl, 5 NaCl, 5 KCl, 5 EGTA, 1 MgCl_2_, 10 HEPES (pH 7.2 with CsOH). Sodium currents were evoked by a single pulse at −10 mV from an holding potential −100 mV, or by a protocol of voltage steps of 10 ms duration ranging from −90 mV to +140 mV in 10 mV increments. The peak decay was fitted with a double exponential function that gave back a slow and a fast time constants (τ_slow_ and τ_fast_, respectively). Conductance-voltage relationship was obtained by normalizing peak I_Na_ to the driving force and expressed as value normalized to maximal I_Na_. Steady-state fast inactivation was studied applying a two steps protocol: a first pulse ranging from −140 to +10 mV (duration 100 ms, increment +10 mV) followed by a test pulse to −10 mV (duration 20 ms, holding −100 mV). Both activation and inactivation curves were fitted with a Boltzmann function y = 1/(1 + exp((V − V_1/2_)/k)) where y is the relative current, V is the membrane potential, V_1/2_ is the half-maximal voltage and k is the slope factor. A two pulses protocol at −10 mV (duration 20 ms) separated by an intermediate step at −100 mV of variable duration (increasing from 0.001 up to 3000 ms) was used to study the recovery from fast inactivation. The entry into slow inactivation protocol had a first inactivating step (P1; at −10 mV, with increasing duration from 50 ms up to 5 s) followed by −100 mV step (90 ms of duration to let the great majority of sodium channels recover from fast inactivation) and a final −10 mV test pulse (P2; duration 15 ms).

Current-clamp patch-clamp recordings on myotubes were performed between the sixth and seventh day of differentiation (T6, T7). The extracellular solution had the following composition (in mM) 140 NaCl, 2.8 KCl, 2 CaCl_2_, 2 MgCl_2_, 10 HEPES, 10 glucose (pH 7.3 with NaOH), while the intracellular one (in mM) 130 KCl, 10 NaCl, 2 MgCl_2_, 5 EGTA, 10 HEPES, 2 K_2_ATP (pH 7.3 with KOH). After obtaining the whole-cell configuration and measured the resting membrane potential,the amplifier was switched to current-clamp mode, the bridge balance compensation was applied and the membrane resting potential was held at −80 mV^29^ by injecting the appropriate current. Action potentials (APs) were evoked through injection of current steps ranging from 0 up to 1000 pA (duration 3 s, increment 50 pA); the minimum injected current that successfully elicited an action potential is defined as the rheobase current. The other parameters analyzed were the action potential overshoot (i.e. the maximum value reached during the uprising phase of the AP), the maximum slope of the uprising phase, as (dV/dt)_MAX_, as well as the action potential duration at 30, 50, 70 and 90% of repolarization (APD_30_, APD_50_, APD_70_ and APD_90_ respectively).

Data were acquired with a Multiclamp 700B amplifier, Digidata 1440A and pClamp 10.3 software, analyzed with Clampfit 10.3 software (all Axon Instruments, Molecular Device) and elaborated with Excel (Microsoft Office) and OriginPro 8 (OriginLab). All data are expressed as mean ± SEM. To determine significance, two-tailed Students t test was used to compare means; p < 0.05 was considered statistically significant and indicated in the results section with *.

### Data availability

The datasets generated and analyzed during the current study are available from the corresponding author on reasonable request.

## Electronic supplementary material

Supplementary Information
