# Supplementary material for: SCN4A as modifier gene in patients with myotonic dystrophy type 2
Source: Sci Rep. 2018 Jul 23;8:11058. doi: 10.1038/s41598-018-29302-z (PMC6056531; doi:10.1038/s41598-018-29302-z)

**SCN4A as modifier gene in patients with myotonic dystrophy type 2**

Anna Binda, Laura V. Renna, Francesca Bosè, Elisa Brigonzi, Annalisa Botta, Rea Valaperta, Barbara Fossati, Ilaria Rivolta, Giovanni Meola, Rosanna Cardani.

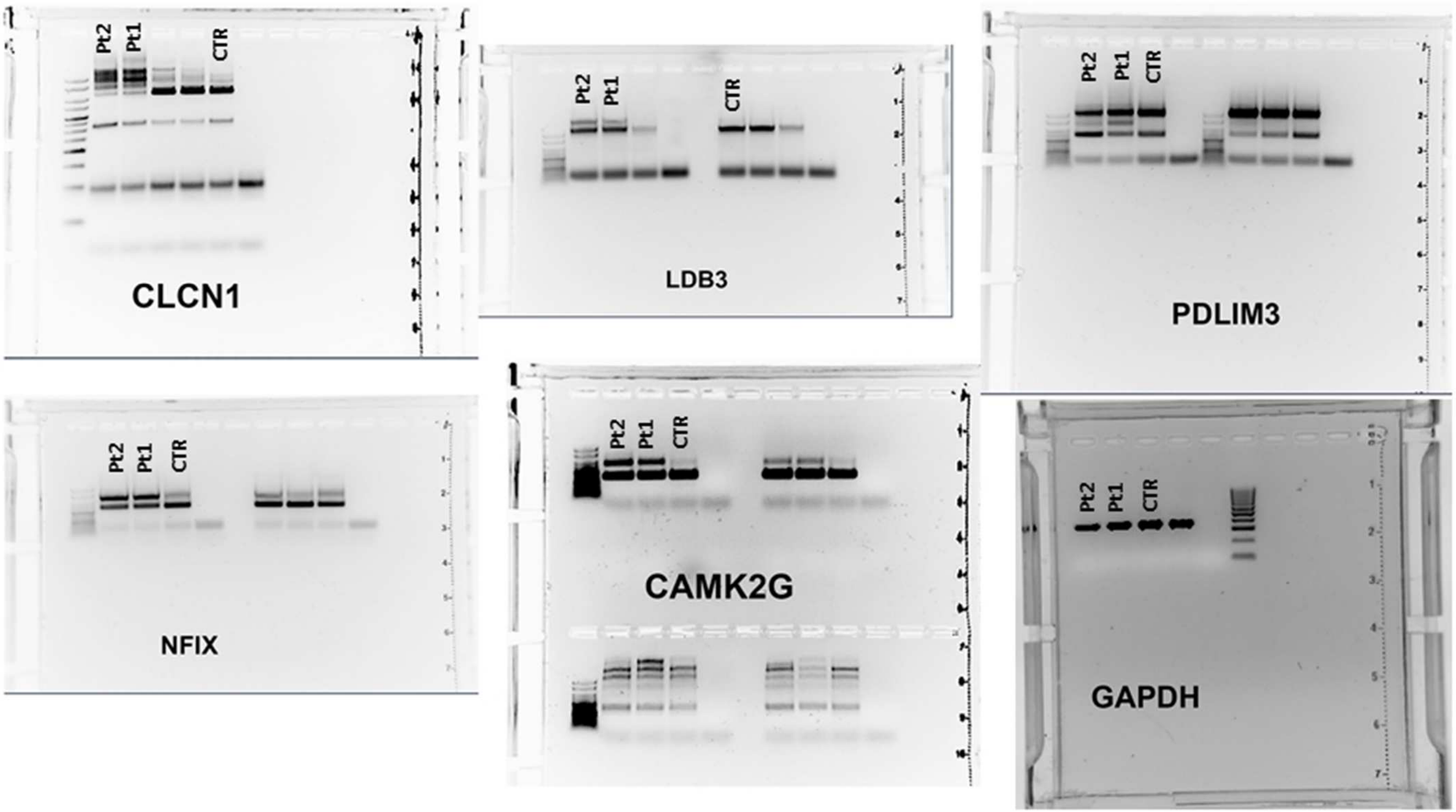

Supplement: Supplementary file 1 — Supplementary Information [file 41598_2018_29302_MOESM1_ESM.pdf]
